# Study of the Effect of Nitric Acid in Electrochemically Synthesized Silicon Nanocrystals: Tunability of Bright and Uniform Photoluminescence

**DOI:** 10.3390/nano12122015

**Published:** 2022-06-10

**Authors:** Alfredo Morales-Sánchez, María Antonia Cardona-Castro, Liliana Licea-Jiménez, Liliana Palacios-Huerta, Antonio Coyopol, Sergio Alfonso Pérez-García, Jaime Alvarez-Quintana, Mario Moreno

**Affiliations:** 1 Electronics Department, Instituto Nacional de Astrofísica, Óptica y Electrónica, Tonantzintla 72840, Puebla, Mexico; mmoreno@inaoep.mx; 2 Centro de Investigación y de Estudios Avanzados del Instituto Politécnico Nacional Unidad Saltillo, Avenida Industria Metalúrgica # 1062, Parque Industrial, Ramos Arizpe 25900, Coahuila, Mexico; 3 Centro de Investigación en Materiales Avanzados S.C., Unidad Monterrey, Parque de Investigación e Innovación Tecnológica (PIIT), Apodaca 66628, Nuevo Leon, Mexico; liliana.licea@cimav.edu.mx (L.L.-J.); alfonso.perez@cimav.edu.mx (S.A.P.-G.); jaime.alvarez@cimav.edu.mx (J.A.-Q.); 4 Instituto Politécnico Nacional, Unidad Profesional Interdisciplinaria de Ingeniería Campus Tlaxcala (UPIIT), Tlaxcala 90000, Tlaxcala, Mexico; lilis.palacios@gmail.com; 5 Centro de Investigación en Dispositivos Semiconductores, Benemérita Universidad Autónoma de Puebla, 14 Sur y Av. San Claudio, Puebla 72000, Puebla, Mexico; acoyopol@gmail.com

**Keywords:** silicon nanocrystals, porous silicon, photoluminescence, electrochemical etching

## Abstract

In this work, we show a correlation between the composition and the microstructural and optical properties of bright and uniform luminescent porous silicon (PSi) films. PSi films were synthesized by electrochemical etching using nitric acid in an electrolyte solution. PSi samples synthesized with nitric acid emit stronger (up to six-fold greater) photoluminescence (PL) as compared to those obtained without it. The PL peak is shifted from 630 to 570 nm by changing the concentration ratio of the HF:HNO_3_:(EtOH-H_2_O) electrolyte solution, but also shifts with the excitation energy, indicating quantum confinement effects in the silicon nanocrystals (Si-NCs). X-ray photoelectron spectroscopy analysis shows a uniform silicon content in the PSi samples that emit the strongest PL. High-resolution transmission electron microscopy reveals that the Si-NCs in these PSi samples are about ~2.9 ± 0.76 nm in size and are embedded in a dense and stoichiometric SiO_2_ matrix, as indicated by the Fourier transform infrared analysis. On the other hand, the PSi films that show PL of low intensity present an abrupt change in the silicon content depth and the formation of non-bridging oxygen hole center defects.

## 1. Introduction

Bulk silicon is unsuitable for optoelectronic and photonic applications due to its indirect bandgap nature [1]. By contrast, silicon nanocrystals (Si-NCs) behave as a quasi-direct transition bandgap material, enhancing an intense room temperature photoluminescence (PL) [1,2,3]. Additionally, it is possible to modulate the PL energy by changing the size of the Si-NCs and/or the surrounding environment because of quantum confinement effects (QCE) [3,4,5]. These characteristics allow for the Si-NCs to be used as functional materials for optoelectronic device applications. For example, Si-NCs embedded in a dielectric matrix have been proved as a functional active material for light-emitting devices and photodetectors [3,6,7,8]. Moreover, Si-NCs have been used to increase the efficiency of Si-based solar cells through the photon down-conversion phenomenon [6,9]. Therefore, Si-NCs could allow for the development of Si-based photonic devices [10].

Multiple techniques have been reported to obtain Si-NCs embedded in a dielectric matrix, the most common being chemical vapor deposition (CVD), either low-pressure (LPCVD) or plasma-enhanced (PECVD) [3,11,12,13,14,15,16,17]. However, in these techniques, the silicon-rich dielectric films require a subsequent thermal annealing process at high temperature to create the Si and SiO_2_ phase separation and thus cause the formation of Si-NCs. The synthesis of Si-NCs at low temperature is required in Si-based technology since high-temperature process steps can affect the operation of the final circuit. On the other hand, the synthesis of functional hybrid inorganic–organic materials has become an interesting subject for the development of flexible electronic technology [18,19]. Si-NCs/polymer composites have also been used as down-converting layers to enhance the efficiency of organic SCs [20]. A simple method to obtain highly PL Si-NCs is the electrochemical etching of a silicon wafer, which forms a porous silicon (PSi) film [18,21]. Si-NCs are embedded in the SiO_x_ (x < 2) pillars that form the PSi film [2,18]. Since PSi is a fragile material, Si-NC powders can be easily extracted through mechanical exfoliation and then embedded in an organic matrix to obtain a flexible membrane [22,23]. However, most of the reported PSi films synthesized by electrochemical etching exhibit red–orange PL [2,18,23,24,25,26] with low intensity, and, in some cases, the PL quenches in time [27,28]. Multiple studies have been devoted to stabilizing, increasing, and modulating the PL of PSi by changing the synthesis parameters [18,22,29,30,31,32]. Some results have demonstrated that the PL intensity of PSi can be increased by the coating of the Si wafer with Ag nanoparticles as a result of the anodic oxidation of silicon in a process known as metal-assisted electrochemical etching (MAECE) [33]. The preparation of luminescent Si-NCs has been also reported when PSi films are irradiated with a 266 nm UV pulsed laser [34]. Post-oxidation of the PSi by rinsing it in hydrogen peroxide (H_2_O_2_) for several minutes, manual milling, and chemical etching of PSi powders are some reported post-processes [35,36,37]. Therefore, highly luminescent PSi films have been achieved only under specific and controlled ambient conditions.

In this work, we show the synthesis of Si-NCs in PSi films using nitric acid in an electrolyte solution. PSi samples synthesized with nitric acid emit a stronger (up to six-fold greater) PL as compared to those obtained without it. Moreover, the PL peak shifts from 630 to 570 nm by changing the concentration ratio of the HF:HNO_3_:(EtOH-H_2_O) electrolyte solution, but it also shifts with the excitation energy, indicating QCE in Si-NCs. A correlation between composition and the microstructural and optical properties of the PSi films was calculated to understand their bright and uniform PL.

## 2. Materials and Methods

The reagents used for the synthesis process of the PSi films were ethanol (EtOH, absolute, ACS, Fermont), nitric acid (HNO_3_, 68–70%, ARISTAR, ACS, VWR Chemicals BDH, Radnor, PA, USA), hydrofluoric acid (HF, 48%, ACS, FERMONT, Monterrey, Mexico), and hydrogen peroxide (H_2_O_2_, 30%, RA ACS, CTR, Monterrey, Mexico). The PSi films were obtained through the electrochemical etching of (100)-oriented p-type silicon substrates with resistivities of 0.1–0.5 Ω-cm, using an electrolyte composed of HF, HNO_3_, and EtOH with deionized (DI) water (HF:HNO_3_:(EtOH-H_2_O)). Prior to etching, Si substrates were ultrasonically cleaned in acetone, ethanol, and DI water for 10 min, respectively, followed by piranha boiling solution. The native oxide was etched by dipping the Si substrates in an HF–DI H_2_O (1:7) solution. The HF:HNO_3_ ratio was fixed at 1:3, while the EtOH-H_2_O (1:1) concentration was changed between 3, 6, 9, and 12, as shown in Table 1. A control sample, without HNO_3_, was also synthetized using an electrolyte composed of HF:(EtOH-H_2_O) (1:3) to observe the effect of the nitric acid. The synthesis was conducted at room temperature and under ambient conditions, applying a current density (J) of 2.39 mA/cm^2^ for 1 h. The as-produced PSi films were rinsed in EtOH to remove organic waste and acids and then immediately immersed in 30% hydrogen peroxide for 10 min to oxidize the PSi films. Finally, the PSi films were dried under ambient conditions.

The PL and PL excitation (PLE) spectra of the PSi films were measured with a Fluoromax 4 from Horiba Jobin Yvon (Horiba Ltd., Kioto, Japan). The samples were excited with different energies (4.96–3.76 eV) and the PL emission signals were collected from 380 to 850 nm with a resolution of 1 nm. A cutoff filter above 370 nm was used to block the light scattered from the source. The PLE spectra were measured from 200 to 400 nm with a resolution of 1 nm, with the detector centered at the wavelength of maximum PL emission of each film. Fourier-transform infrared (FTIR) spectroscopy measurements were obtained with a Bruker model Vector 22 spectrometer (Ettlingen, Germany). The silicon and oxygen content of the PSi films was analyzed (in-depth profile) by X-ray photoelectron spectroscopy (XPS) with an Escalab 250Xi from Thermo Scientific (West Sussex, England). A JEOL JEM 2200 (Jeol Ltd., Tokyo, Japan) high-resolution transmission electron microscope (HRTEM) was used to observe and determine the size of the Si-NCs. PSi powder with embedded Si-NCs dispersed in 1-propanol was drop-casted onto a carbon-coated TEM grid for this analysis.

## 3. Results and Discussion

**Figure 1:**
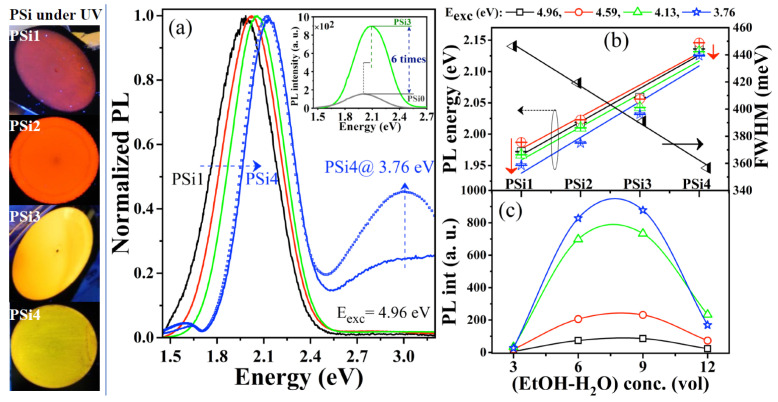
Images of PSi films under UV excitation (left): (**a**) normalized PL spectra; (**b**) PL peak energy and FWHM; and (**c**) PL intensity for different energy excitations of PSi films and as function of the EtOH-H_2_O concentration in the HF:HNO_3_:(EtOH-H_2_O) electrolyte. Labels PSi1-PSi4 are also indicated for clarity in their respective EtOH-H_2_O concentrations. Inset in (**a**) shows a comparison between PSi0 and PSi3 films. Lines in (**c**) are for eye-guide.

Figure 1a shows the normalized PL spectra of the PSi films synthesized with the different HF:HNO_3_:(EtOH-H_2_O) electrolytes and excited with 4.13 eV. All PSi films emit intense and uniform PL, as shown in the pictures on the left side of Figure 1. A comparison of the PL of the PSi0 (without nitric acid) and PSi3 samples is shown in the inset of Figure 1a. As we can see, the PL intensity of PSi3 is almost six times higher as compared to PSi0. Therefore, stronger PL and a blueshift of the peak are obtained with the presence of HNO_3_ in the electrolyte. The PL peak also blueshifts as the EtOH-H_2_O (1:1) concentration increases from 3 (PSi1 at 1.97 eV) to 12 (PSi4 at 2.13 eV), while the PL spectrum becomes narrower, as observed by the full width at half maximum (FWHM) value in Figure 1b.

Both the shift of the PL peak (for any excitation energy) and its width (FWHM) exhibit a quasi-linear behavior as a function of the EtOH-H_2_O concentration, as observed in Figure 1b. The blueshift of the PL peak observed in the PSi films can be related to the QC model in Si-NCs, while the decrease in FWHM can be related to a narrow distribution of the Si-NC sizes. This behavior is similar to that observed by X. Wen et al. [38], who studied size-selected Si-NCs. They observed PL blueshift and a narrower PL band as the Si-NC size decreased from 6.2 to 3.8 nm and from 3.8 to 2.5 nm.

Figure 1b shows that the PL peak also blueshifts as the excitation energy increases, which implies QC effects through the Si-NC size selection [38,39]. Nevertheless, this energy shift of the PL peak becomes smaller as the EtOH-H_2_O concentration increases, indicating a possible narrower Si-NC size distribution or the presence of a preferentially luminescent Si-NC surface defect [4,38]. The PL intensity is also affected by the EtOH-H_2_O concentration in the electrolyte solution, as observed in Figure 1c. The most intense PL is obtained when the EtOH-H_2_O concentration is six (PSi2) or nine (PSi3). Moreover, the maximum emission is observed with an excitation energy of 3.76 eV in all the PSi samples.

**Figure 2:**
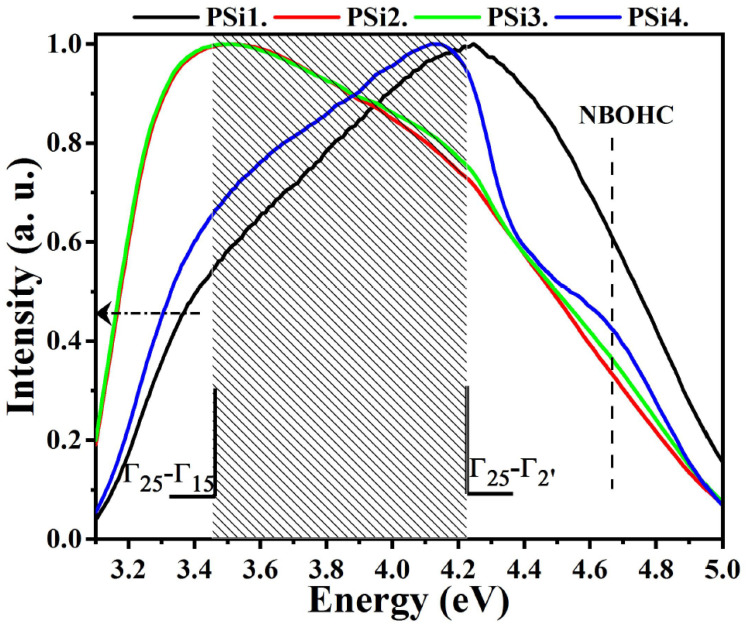
PLE spectra of PSi films measured at their maximum PL peak energy.

PLE measurements were performed to understand the origin of the PL bands. Figure 2 shows the PLE spectra of the PSi films, normalized to their maximum intensity. All the PSi samples display a broad band extending from 3.1 to 5 eV, with a maximum near the direct band-gap energies of bulk crystalline silicon: Γ_25_→Γ_15_ at ~3.4 eV and Γ_25_→Γ_2′_ at ~4.2 eV [40,41,42], indicating that absorption is dominated by direct transitions.

It has been reported that QC effects influence the edge of the PLE band [3,41]. As we can see, the PLE band edge redshifts as the electrolyte becomes more diluted with EtOH-H_2_O from PSi1 to PSi4. Moreover, the largest redshift is observed for the PSi2 and PSi3 samples, compared to that for the PSi1 sample. It has been reported that the energy of indirect transitions increases when the Si-NC size decreases, with an apparent redshift in the direct transition energy [43]. This redshift and the enhanced PL intensity in the direct transition have been related to the presence of Si-NCs with an average size near to 2 nm [43]. Therefore, the redshift observed in the PSi samples in this work implies that the QC effects in direct transitions may be the result of the small Si-NCs that are preferentially formed when a diluted electrolyte is used, especially for the PSi2 and PSi3 samples.

The PLE signals extend up to 5 eV for all the PSi samples, but a shoulder at about 4.7 eV is well defined for the PSi4 sample and the most intense signal in this high-energy region is obtained for PSi1. The non-bridging oxygen hole center (NBOHC) has been related to absorption and emission bands at about 4.8 eV and 1.9–2 eV, respectively [3,44,45]. The main PL band for the PSi4 sample remains at almost the same energy, at about 2.1 eV, when it is excited with different energies (see Figure 1b). It is well known that the PL peak of luminescent defects remains at the same energy for excitation with different energies [35,45]. Additionally, the most relevant effects at the Si-NC/SiO_2_ interface are produced when the Si-NC sizes are maintained below 2 nm [4]. Under these dimensions, the surface states strongly influence the optical properties, overcoming the QC effects. Therefore, the presence of NBOHC defects at the Si-NC surfaces or their vicinities [43,46] can affect the PL spectrum of the PSi4 film. The presence of this defect can explain the theoretical and experimental studies which indicate that there exists a limit of the PL energy below 2.1 eV for Si-NCs smaller than 2 nm [36,38]. Indeed, a quenching of the PL intensity has also been observed at that energy limit, such as in this work (see Figure 1c). On the other hand, as observed in Figure 1a, the PSi4 sample emits an additional UV/blue PL band, located at 3 eV, which is not present in the other PSi films (see Supplementary Materials, Figure S1). Moreover, this band becomes more intense when the excitation energy is reduced to 3.76 eV. Therefore, the appearance of this UV/blue PL band can be explained by the QC effects in Si-NCs with sizes below 2 nm, as proposed in [43,47].

**Figure 3:**
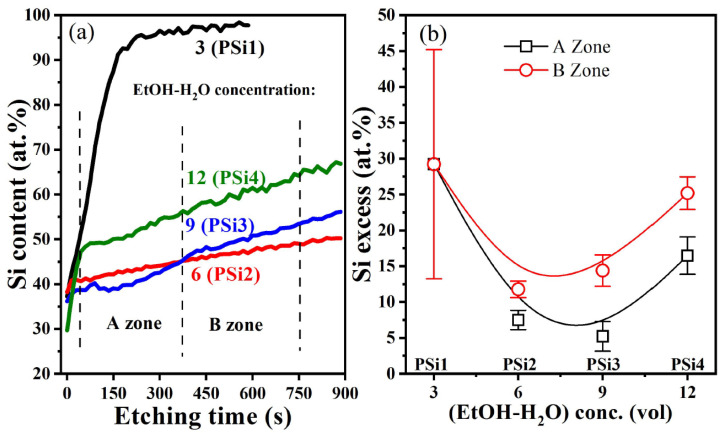
Si content (**a**) in the depth profile and (**b**) as a function of the EtOH-H_2_O concentration for the different PSi films. Labels PSi1-PSi4 are also indicated for clarity in their respective EtOH-H_2_O concentrations for clarity.

XPS measurements in the depth profile were performed in all the PSi samples to determine their silicon and oxygen content; a representative depth profile spectrum for the PSi3 sample is shown (see Supplementary Materials, Figure S2) together with the Si2p signal for all depth profiles. For clarity, Figure 3a only shows the Si2p profile within the PSi films.

When the HF:HNO_3_:(EtOH-H_2_O) electrolyte is highly concentrated (low EtOH-H_2_O, PSi1 sample), the silicon content increases abruptly from 36 at. % at the surface to 80 at. % at the short etching time. It is clearly seen from the Si profiles that PSi1 is the thinnest sample. The thickness, as measured by scanning electron microscopy (SEM, see Figure S3 in Supplementary Materials), is about 0.377 ± 0.027, 3.62 ± 0.130, 2.67 ± 0.15, and 5.79 ± 0.18 μm for the PSi1, PSi2, PSi3, and PSi4 films, respectively. As the electrolyte is diluted with EtOH-H_2_O (PSi2 and PSi3), the silicon content reduces and becomes more uniform in depth, as compared to the PSi1 sample. Nevertheless, two zones can be appreciated where the Si content changes in the PSi2 sample respect to PSi3.

It has been described that the chemical etching of Si using HF-HNO_3_-H_2_O solutions can be considered as an electrochemical process, which is initiated by a chemical reaction. First, cathodic and anodic sites are formed on the Si surface. In the anode, the dissolution of Si occurs, while in the cathode reaction, the reduction of HNO_3_ occurs, causing the injection of holes (h+) towards the Si valance band [48]. Therefore, these h+ can be considered as a by-product of Si attack or dissolution and are located at the Si wafer/electrolyte solution interface. In this case, in an electrolyte solution of 1:3:3 HF:HNO_3_:(EtOH-H_2_O) (PSi1), an excess of holes was created that instantly covered the Si surface, causing the direct attack of the minority molecules of HF on Si almost instantaneously. This fast (immediate) etching speed can explain the lower thickness (~380 nm), the abrupt Si content, and the higher FWHM value of the PL band (see Figure 1b). This drastic decrease in the etching rate of the PSi1 film coincides with observations made by [48,49], who attributed this behavior (at HNO_3_ concentrations above 40% in HF-HNO_3_ solutions) to an etching regime controlled by diffusion and not by the chemical reaction of the reactive species of the system. Previously, it has been described [49] that the thickness of the PSi film depends on the diffusion of reagents and reaction products between the electrolyte solution and the etched Si surface. In an electrolyte solution of 1:3:3 HF:HNO_3_:(EthOH-H_2_O) (PSi1), the surface of the Si wafer was instantly saturated with the reaction products; in this case, it may have been the voids (without the formation of oxides) or the water-soluble compound H_2_SiF_6_ which stabilized and formed a layer of reaction products that prevented the diffusion of reagents towards the Si wafer, obstructing the growth of the PSi1 film and transferring the chemical reaction of the etching to the electrode, where it carried out the electro-oxidation of ethanol. On the contrary, the PSi4 film, with an electrolyte solution of 1:3:12 HF:HNO_3_:(EtOH:H_2_O), obtained the greatest thickness (5.8 µm on average) and the lowest FWHM value of PL (Figure 1b), which experimentally reflected a slow etching rate dominated by the etching chemical reaction.

Figure 3b shows the average silicon excess, above the 33.3 at. % of stoichiometric silicon oxide, within the PSi films. In the A zone, the Si content is about 9.6 ± 1.28 at. % and 7.5 ± 1.96 at. %, while in the B zone it is about 13.7 ± 1.08 at. % and 16.2 ± 2.08 at. % for the PSi2 and PSi3 films, respectively. In any case, the average silicon content is quite similar for both PS films, being 11.65 ± 2.87 at. % and 11.81 ± 6.15 at. % for the PSi2 and PSi3 films, respectively. This effect explains why these PSi films exhibit similar optical properties. Moreover, the lowest and uniform (in depth) Si excess can be related to the highest PL observed in these samples, since a uniform and low Si excess indicates a narrow size distribution and smaller Si-NCs. By contrast, the non–uniform and abrupt increase in the Si excess in the PSi1 film could promote the formation of larger Si-NCs with a broad size distribution, emitting the lowest PL intensity.

**Figure 4:**
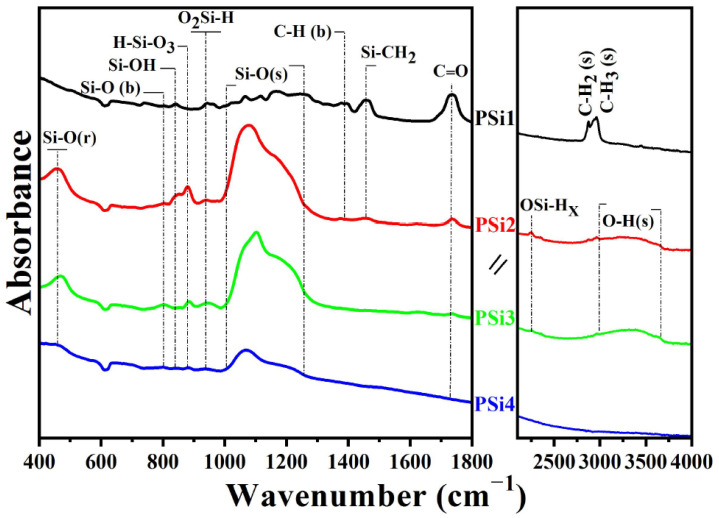
IR absorbance spectra of PSi films.

Figure 4 shows the FTIR spectra of the PSi films. The higher oxygen concentration in the PSi2, PSi3, and PSi4 samples is clearly observed by the presence of a well-defined band at about 1072 cm^−1^, with a shoulder at about 1160 cm^−1^, and two minor bands at 460 cm^−1^ and 800 cm^−1^, corresponding to Si–O bonding for the stretching (s), asymmetric stretching (as), rocking (r), and bending (b) vibration modes, respectively [50,51]. Indeed, the PSi2 and PSi3 samples which emit the highest PL exhibit the strongest O-related IR absorption bands. Furthermore, the Si–O–Si (s) band for PSi2 and PSi3 shifts to higher wavenumbers in comparison with the PSi4 sample, where it is placed at 1072 cm^−1^. The displacements of the Si–O stretching IR peak towards higher wavenumbers and the increase in their absorption intensity, as compared to the PSi4 sample, are attributed to the formation of a dense and stoichiometric SiO_2_ matrix [50,52]. Therefore, the Si-NCs formed in the PSi2 and PSi3 films are embedded in a stoichiometric silicon oxide film. On the other hand, the robust shoulder at about 1160 cm^−1^ has been related to the presence of a higher amount of Si-NCs and a higher tension caused by the phase separation between the Si-NCs and the SiO_2_ matrix [53].

IR bands related to the presence of hydrogen atoms in PSi2 and PSi3 are also observed. These IR peaks are related to Si_3_–Si–H wagging (840 cm^−1^), O_3_–Si–H bending (880 cm^−1^), Si–OH (948 cm^−1^), Si–H stretching of the H–Si–O_3_ configuration (2256 cm^−1^), water (broad band at 3300 cm^−1^), and O–H stretching (3650 cm^−1^) [39,52,54]. The couple of IR peaks at 840 cm^−1^ and 880 cm^−1^, as observed in the PSi2 and PSi3 samples, are related to the doublet (wagging–bending) bonds of the (Si–H_2_)n groups and they indicate that there is a small amount of hydrogen bonded in the form of a dihydride [50]. It is well known that hydrogen passivates non-radiative defects in luminescent SiO_x_ (x < 2) films [55]. Therefore, the incorporation of higher oxygen and hydrogen concentrations on the surface of the Si–NCs embedded in the PSi2 and PSi3 films enhances their high PL intensity. On the other hand, the PSi1 sample, which emits the lowest PL intensity compared with the other PSi films, exhibits IR bands related to Si_3_–Si–H wagging (840 cm^−1^), Si–OH (948 cm^−1^), and Si–O stretching with very low intensity. Furthermore, this sample exhibits IR bands related to carbon, such as C=O stretching (1736 cm^−1^), C–Hx bending (1355–1496 cm^−1^), and C–Hx stretching (2832–3000 cm^−1^) [56,57]. In fact, the most intense IR bands in the PSi1 sample are the C=O and both C–H_x_ (b and s) bands, which are related to the formation of acetaldehyde, which is an undesired sub-product of a polluting nature produced by the ethanol electro-oxidation [58,59,60]. The PSi1 and PSi4 samples exhibit lower amounts of Si–O–Si and Si–H bonds. Si–H and Si–O–Si bond deficiency has been related to the formation of E‘ (an unpaired electron in a Si dangling bond) and nonbridging oxygen hole center (NBOHC, ●O–Si≡) defects in silicon-implanted SiO_2_ films [61]. This effect agrees with the presence of NBOHC defects in the PSi samples from this work and it is confirmed by the PLE peak at 4.7 eV (see Figure 2). 

**Figure 5:**
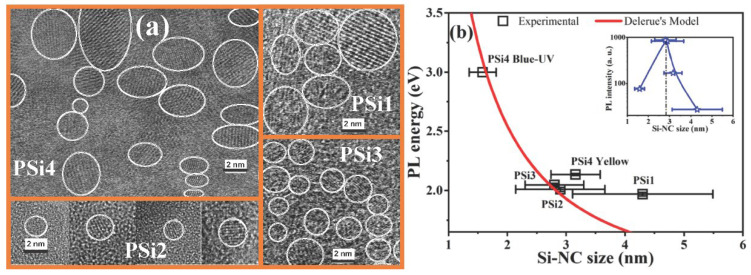
(**a**) HRTEM images of PS films and (**b**) PL energy as a function of the Si-NC. Inset in (**b**) shows the PL intensity for different Si-NC size.

HRTEM images were obtained to confirm the presence of Si-NCs within the different PSi films. The different lattice fringes observed in the TEM images, shown in Figure 5a, indicate the formation of Si-NCs with different sizes depending on the EtOH-H_2_O concentration in the electrolyte. Si-NCs with a broad size distribution and a mean size of about ~4.3 ± 1.19 nm are observed for the PSi1 film, while the PSi2 and PSi3 samples, which emit the strongest PL, have Si-NCs with a narrower size distribution and with mean sizes of ~2.9 ± 0.76 nm and ~2.8 ± 0.5 nm, respectively. Two main Si-NC size distributions were obtained for the PSi4 film, with average sizes of ~3.16 ± 0.42 nm and ~1.58 ± 0.23 nm. The presence of these two size distributions in PSi4 could explain the two PL bands observed in this film as the excitation energy decreases. On the one hand, the PL band at 2.13 eV can be related to the presence of NBOHC defects, while that PL band at 3 eV is originated by the Si-NCs with sizes of about 1.58 nm, as observed in Figure 5b. As we can observe, Delerue´s model agrees with the experimental PL energy of 3 eV [62]. On the other hand, the presence of NBOHC can explain the theoretical and experimental studies which indicate that there exists a limit of the PL energy below 2.1 eV for Si-NCs smaller than 2 nm. In fact, the PSi1 sample, which also exhibits NBOHC defects, as observed in the PLE spectra, emits at a higher energy than that theoretically expected.

On the contrary, the experimental data for the PL energy of the PSi2 and PSi3 samples agree with those expected by Delerue´s model. Moreover, the presence of Si-NCs with a narrow size distribution in these PSi films enhances the QC effects, resulting in their strongest PL, as observed in the inset of Figure 5b. In this graph, it is observed that the maximum PL is obtained with Si-NCs of about 2.9 nm in size. Similar results have been reported with 2.6 nm-sized Si-NCs embedded in silicon-rich oxide films deposited by LPCVD [63]. Additionally, a quenching of the PL was obtained for Si-NCs bigger than 4 nm. Therefore, the PSi2 and PSi3 films offer alternatives as luminescent materials for their incorporation in a polymeric matrix that acts as a PL-flexible membrane and can be applied to optoelectronic devices.

## 4. Conclusions

Si-NCs in PSi films were synthesized by electrochemical etching, using nitric acid in the electrolyte solution. Stronger PL is obtained with nitric acid, as compared to that obtained without it. All PSi films emit intense and uniform PL. Si-NCs with sizes between 1.58 and 4.3 nm were obtained, depending on the electrolyte concentration. The strongest PL was obtained with a Si-NC size of about 2.9 ± 0.76 nm and related to QC effects, as indicated by Delerue’s model. Furthermore, this intense PL is related to the incorporation of higher oxygen and hydrogen concentrations on the surfaces of the Si–NCs, as indicated by FTIR and XPS studies. The analysis of the PLE spectra indicates that the presence of NBOHC defects influence the PL energy and intensity of the PSi samples with the largest and smallest Si-NCs (4.3 nm and 1.5 nm). The presence of this defect can explain the theoretical and experimental studies which indicate that there exists a limit of the PL energy below 2.1 eV for Si-NCs smaller than 2 nm.

## Figures and Tables

**Table 1:** Samples description.

Sample	Electrolyte Ratio			J (mA/cm^2^)	Time (min)
	HF	HNO_3_	EtOH:H_2_O (1:1)		
PSi0	1	0	3	2.39	60
PSi1	1	3	3		
PSi2			6		
PSi3			9		
PSi4			12		

## Data Availability

Not applicable.

## Supplementary Materials

The following supporting information can be downloaded at: https://www.mdpi.com/article/10.3390/nano12122015/s1, Figure S1: Normalized PL spectra of PSi films for energy excitation of (a) 4.96 eV, (b) 4.59 eV, (c) 4.13 eV and (d) 3.76 eV; Figure S2: Atomic concentration of Si2p, O1s and N1s in depth profile for the PSi3 sample. Inset shows all the signals related to the Si2p signal of the different etching levels; Figure S3: Cross-section SEM micrographs of the PSi films as a function of the EtOH-H_2_O concentration in the HF:HNO_3_:(EtOH-H_2_O) electrolyte. Labels PSi1-PSi4 are also indicated for clarity in their respective EtOH-H_2_O concentration.
