# Supplementary material for: Study of the Effect of Nitric Acid in Electrochemically Synthesized Silicon Nanocrystals: Tunability of Bright and Uniform Photoluminescence
Source: Nanomaterials (Basel). 2022 Jun 10;12(12):2015. doi: 10.3390/nano12122015 (PMC9229415; doi:10.3390/nano12122015)
Supplement: Supplementary file 1 [file nanomaterials-12-02015-s001.zip › nanomaterials-1736582-supplementary.pdf]

nanomaterials

MDPI

Supplementary Materials

Study of the Effect of Nitric Acid in Electrochemically Synthesized Silicon Nanocrystals: Tunability of Bright and Uniform Photoluminescence

Alfredo Morales-Sánchez [1,*], María Antonia Cardona–Castro [2,*], Liliana Licea-Jiménez [3], Liliana Palacios-Huerta [4], Antonio Coyopol [5], Sergio Alfonso Pérez-García [3], Jaime Alvarez-Quintana [3] and Mario Moreno [1]

1 Electronics Department, Instituto Nacional de Astrofísica, Óptica y Electrónica, Tonantzintla 72840, Puebla, Mexico; mmoreno@inaoep.mx
2 Centro de Investigación y de Estudios Avanzados del Instituto Politécnico Nacional Unidad Saltillo, Avenida Industria Metalúrgica # 1062, Parque Industrial, Ramos Arizpe 25900, Coahuila, Mexico
3 Centro de Investigación en Materiales Avanzados S.C., Unidad Monterrey, Parque de Investigación e Innovación Tecnológica (PIIT), Apodaca 66628, Nuevo Leon, Mexico; liliana.licea@cimav.edu.mx (L.L.-J.); alfonso.perez@cimav.edu.mx (S.A.P.-G.); jaime.alvarez@cimav.edu.mx (J.A.-Q.)
4 Instituto Politécnico Nacional, Unidad Profesional Interdisciplinaria de Ingeniería Campus Tlaxcala (UPIIT), Tlaxcala 90000, Tlaxcala, Mexico; lilis.palacios@gmail.com
5 Centro de Investigación en Dispositivos Semiconductores, Benemérita Universidad Autónoma de Puebla, 14 Sur y Av. San Claudio, Puebla 72000, Puebla, Mexico; acoyopol@gmail.com
* Correspondence: alfredom@inaoep.mx (A.M.-S.); antonia.cardona@cinvestav.edu.mx (M.A.C.-C.)

1. Photoluminescence at different excitation energy

Figure S1 shows the PL spectra of PSi films synthesized with the different HF:HNO_3:(EtOH-H_2O) electrolyte excited at different energy. As we can observe, the main PL peak blueshifts as the EtOH-H_2O (1:1) concentration increases from 3 (PSi1) to 12 (PSi4) for all the excitation energies. Also the full width at half maximum (FWHM) of the PL band decreases as the EtOH-H_2O (1:1) concentration increases.

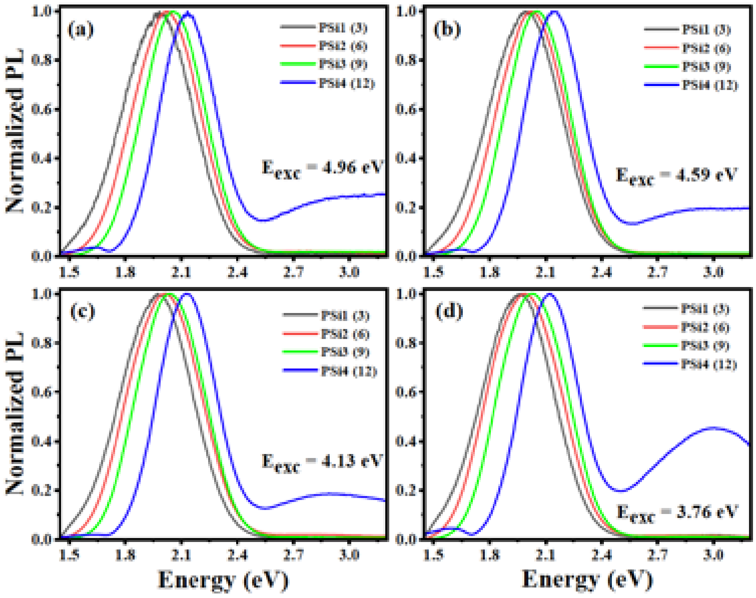

Figure S1. Normalized PL spectra of PSi films for energy excitation of (a) 4.96 eV, (b) 4.59 eV, (c) 4.13 eV and (d) 3.76 eV.

PSi4 sample emits an additional UV/blue PL band located at about 3 eV that is not present in the other PSi films. This PL band appears for all the excitation energies, being the most intense when the excitation energy reduces to 3.76 eV (Figure S1d). The shift of the PL peak (for any excitation energy) and its width (FWHM) as a function of the EtOH-H_2O concentration, is reported in Figure 1b of the manuscript. Also, the PL intensity of each PSi film is reported within the manuscript as Figure 1c.

2. XPS spectra: Si2p, O1s and N1s profiles

The corresponding XPS spectrum in depth-profile of PSi3 sample with its Si2p signals (inset Figure S2) corresponding to all the signals taken in each of the etching level are shown in Figure S2. As we can see, at the surface the oxygen content is larger than the Si. The Si content increases (O reduces) as the etching depth increases. The Si content in depth-profile for the different PSi1-PSi4 films are shown in Figure 3 of the manuscript.

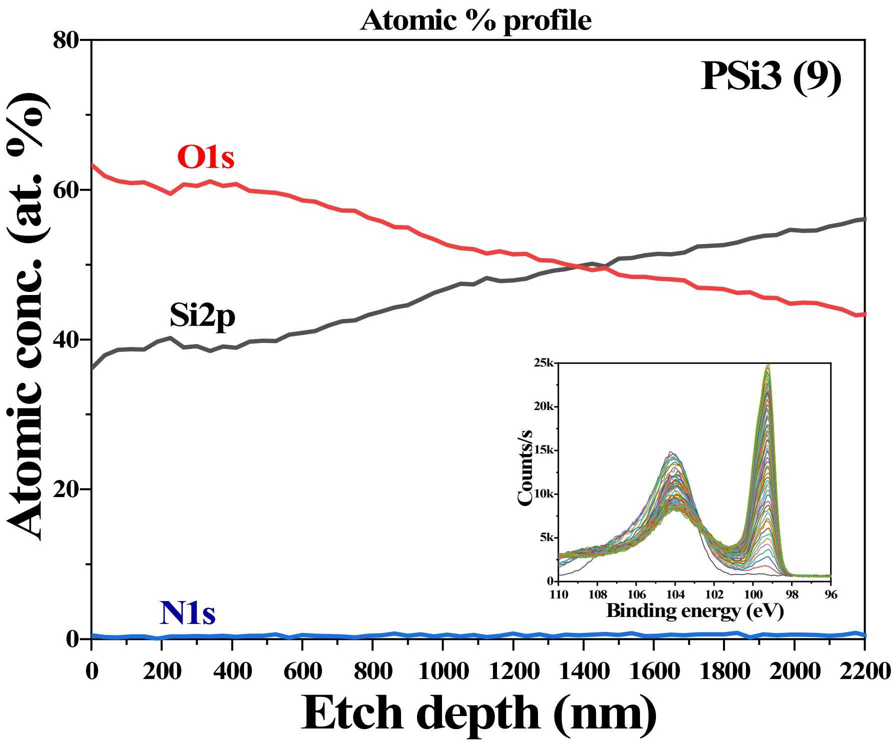

Figure S2. Atomic concentration of Si2p, O1s and N1s in depth profile for the PSi3 sample. Inset shows all the signals related to the Si2p signal of the different etching levels.

3. SEM micrographs: thickness of the PSi films

The thickness of the different PSi films was measured through scanning electron microscopy (SEM) micrographs. The PSi samples were analyzed in cross-section view. Figure S3 shows representative SEM micrographs where we can see that PSi1 is the thinnest film (Figure S3a). Then, the PSi films becomes thicker as the EtOH-H_2O concentration increases, being the PSi4 film the thickest. The analysis on different SEM micrographs for each PSi film shows that the thickness is about 0.377 ± 0.027, 3.62 ± 0.130, 2.67 ± 0.15 and 5.79 ± 0.18 µm for PSi1, PSi2, PSi3 and PSi4 films, respectively.

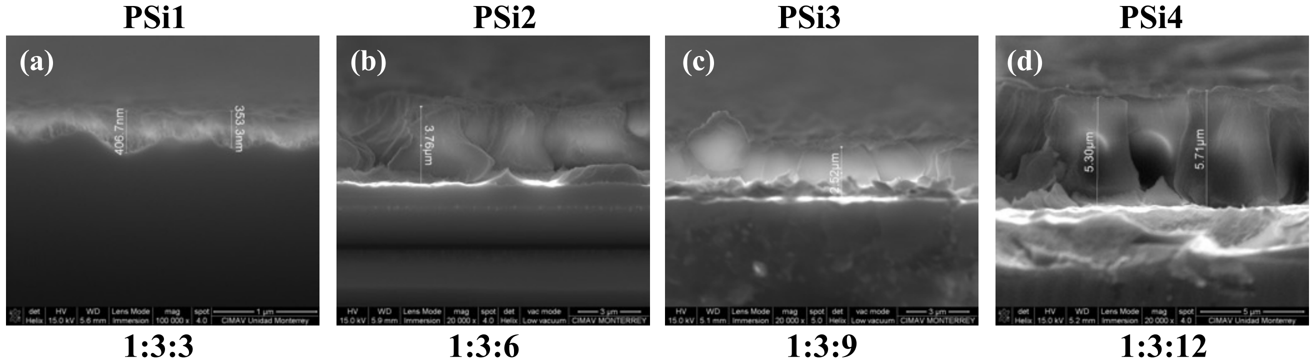

Figure S3. Cross-section SEM micrographs of the PSi films as a function of the EtOH-H_2O concentration in the HF:HNO_3:(EtOH-H_2O) electrolyte. PSi1-PSi4 labels are also indicated for clarity in their respective EtOH-H_2O concentration.
